# Suspected cyanide toxicity in cattle associated with ingestion of laurel - a case report

**DOI:** 10.1186/s13620-021-00188-0

**Published:** 2021-03-24

**Authors:** Aideen Kennedy, Audrey Brennan, Celine Mannion, Maresa Sheehan

**Affiliations:** 1 Kilkenny Regional Veterinary Laboratory, Department of Agriculture, Food and the Marine, Kilkenny, Ireland; 2 Hawkfield Veterinary Clinic, Co. Kildare Hawkfield, Newbridge, Ireland; 3 Biochemistry/Toxicology, Pathology Division, Department Agriculture, Food and the Marine, Backweston Campus, Co. Kildare Celbridge, Ireland

**Keywords:** Cyanide, Laurel, Toxicity, Bovine

## Abstract

**Background:**

Cyanide is one of the most rapidly acting toxins affecting cattle, with poisoning typically occurring following ingestion of cyanogenic plants. Laurel *(Prunus laurocerasus)*, is one such potentially toxic cyanogenic plant. This case report details fatalities in an Irish herd following the ingestion of laurel and aims to raise awareness of the potential risk that access to laurel hedges poses to farm animals.

**Case presentation:**

Over a twelve-day period, the death occurred of 36 dairy-cross weanlings; the majority (22 weanlings) died over a two-day period. Two days following entry to a field bounded by a laurel hedge, the weanlings displayed signs of lethargy and profuse green diarrhoea. In the majority of animals there was a limited response to treatment with antimicrobials, vitamin B complex and fluid therapy. Recumbency and death ensued. Cyanosis was noted terminally. Two weanlings were submitted for post mortem examination. Laurel leaves were identified in the rumen contents of one weanling. Post mortem findings and additional test results on cohort animals suggested a number of pathological processes may have been involved in the animals, possibly complicating/exacerbating the effects of laurel ingestion. However, cyanide was considered a factor in a least some of the casualties and arrangements were made to test for cyanide on blood samples from a random selection of seven cohort animals. Although collected one week after exposure to the laurel hedge, toxic cyanide levels were recorded in a sample from one animal, which subsequently died.

**Conclusions:**

The large fatality rate serves as a timely reminder to include plant poisoning as a differential diagnosis when dealing with large numbers of rapid fatalities. Failure to thoroughly examine rumen contents and collect a detailed history in this case, could easily have allowed death to be attributed to other causes and the involvement of cyanide toxicity to be missed. In cases of individual or group fatalities, history is invaluable and recent entry to new grazing areas or any potential diet change or access to garden plants should be thoroughly investigated.

## Background

Several garden plant species are potentially toxic if ingested by farm animals. Laurel *(Prunus laurocerasus)*, a common garden hedge, is one such potentially toxic cyanogenic plant [1]. Cyanide, the lethal agent of cyanogenic plants, prevents haemoglobin in erythrocytes from releasing oxygen to the tissues, with animals ultimately dying of anoxia [2]. In Ireland, individual animal fatalities relating to ingestion of various toxic plants are not uncommon, however group poisonings are less frequently reported [3]. To the authors’ knowledge this is the first case report detailing multiple fatalities in an Irish herd following ingestion of laurel plant material. This case report aims to raise awareness of the potential risk that access to laurel hedges poses to farm animals, and to act as a reminder to include plant poisoning as a differential diagnosis when dealing with multiple rapid fatalities.

Cyanide is one of the most rapidly acting toxins affecting cattle [4], with poisoning typically occurring following ingestion of cyanogenic plants. There are over 1000 species of cyanogenic plants, but only a small number of these contain sufficient concentrations of cyanogenic glycoside compounds to release fatal doses of hydrogen cyanide [5]. Within the plant cells, the cyanogenic glycosides and the enzymes that convert them to free cyanide gas reside in different locations. When the integrity of the plant cell is compromised by chewing, crushing, wilting or freezing, the enzymes can unite with the cyanogenic glycosides and generate hydrogen cyanide (HCN) [6].

The cyanogenic potential of plants varies with species, soil fertility, weather and stage of plant growth [7]. Environmental stressors are an important risk in acute cyanogenic glycoside poisoning [2]. Relevant environmental factors include periods of drought that slow growth, favouring the formation of cyanogenic compounds in the leaves, high soil nitrogen: phosphorous ratios, soil phosphorous deficiencies and low soil sulphur, which decreases the detoxification of cyanogenic glycosides to thiocyanates within plants [8–10]. Rapidly growing young plants or regrowth on plants after cutting can also have high glycoside content. Frost can allow conversion to HCN within the plant, and grazing of cyanogenic plants after frost can be extremely dangerous. Herbicide can increase the palatability of plants, potentially increasing the risk of consumption [4, 9]. Plant species of veterinary importance include; *Sorghum* species, *Linum* species (linseeds and flaxes), *Sambuchus nigra* (elderberry), *Hydrangea* species and members of the *Prunus* genus (Apricots, peaches and cherry laurels- the cyanogenic plant ingested in this case) [7, 8, 10].

Ruminants are more susceptible to poisoning than monogastric species due to the presence of enzymes in the rumen microflora that facilitate rapid hydrolysis of the cyanogenic glycosides [9]. In addition, the higher pH in the rumen compared with monogastric stomach pH favours the rate of conversion releasing more HCN [5, 10]. As HCN is liberated in the rumen it is absorbed into the bloodstream. Tight complexes are formed between free CN^−^ and oxidised iron (Fe^3+^) in cytochrome complexes. This leads to inhibition of mitochondrial electron transport [5, 11] and the affected animals die of anoxia [2]. With low levels of exposure, cattle and sheep are capable of detoxifying cyanide to thiocyanate in the liver and excreting the metabolite in the urine [8, 12]. However, when large quantities are absorbed rapidly, cyanide is highly potent and the body’s detoxification mechanisms are overwhelmed with death ensuing often in less than two hours [4, 8]. Excitement can be initially displayed by affected animals, followed by tachypnoea and tachycardia. Exhaled breath may smell of bitter almonds. Lacrimation, salivation and voiding of faeces and urine may occur. Generalised spasms and coma can ensue before death. Mucous membranes are often bright red, but can become cyanotic terminally [4, 13]. Histological and gross changes are not consistently seen post mortem. Venous blood is classically bright red but this colour fades rapidly after death [8, 13]. Agonal haemorrhages may be seen. There may be congestion in the lungs and dyspnoeic-type haemorrhages in the tracheal mucosa [10, 12]. Ecchymoses of the epicardium can also be seen. Identification of appropriate plant material in the rumen may be a key finding [1, 14]. Due to the volatility and rapid breakdown of HCN, confirmatory detection can be a difficult and tedious procedure [15, 16] and can lead to false negative test results. Delays in sample processing may further affect the sensitivity of the test [16].

## Case presentation

The following case report details the death of 36 weanlings in a herd over twelve days; the majority (22 weanlings) died over a two-day period. The farm enterprise consisted of 51 dairy-cross weanlings. These animals ranged in age from five to seven months. These weanlings were grazed outdoors and fed calf concentrate once per day. They had *ad lib* access to mains supply water. The weanlings had been purchased several months earlier and the farmer reported that they had been in good health since purchase. A rotational grazing system was in operation on the farm.

Two days following entry to a field on the grazing rotation, a high proportion of weanlings developed clinical signs of lethargy and a profuse green diarrhoea. On developing the clinical signs, the animals were removed from this field to another nearby field. Grass clippings from several neighbouring lawns were identified at the margins of the original field and ruminal acidosis was suspected. There was deterioration in clinical signs and the animals were group housed indoors on straw bedding for veterinary treatment. The weanlings were treated with intravenous fluids and oral fluids with sodium bicarbonate, and sulfadimidine powders were also administered orally. A combined solution for injection of penicillin and streptomycin was administered intramuscularly. Injections of Vitamin B complex were also administered. During treatment the attending veterinarian noticed a degree of cyanosis in the muzzles and mucous membranes of some calves. There was limited response to treatment; a number of weanlings rapidly deteriorated and became moribund before dying. Over the course of one weekend 22 animals died. In an effort to reach a definitive diagnosis and aid direction of treatment of remaining weanlings, an eight-month-old animal (weanling 1) was referred for post mortem examination (PME) to the Department of Agriculture Food and the Marine Regional Veterinary Laboratory (RVL) in Kilkenny (main PME findings below).

Given the rapidity of clinical signs and the numbers affected, the pathologist advised examination of the field for toxic substances while awaiting laboratory results. In addition, a farm visit was organised for the following day to be attended by the farmer, attending veterinarian and RVL veterinary research officers.

### Farm Visit

**Fig. 1:**
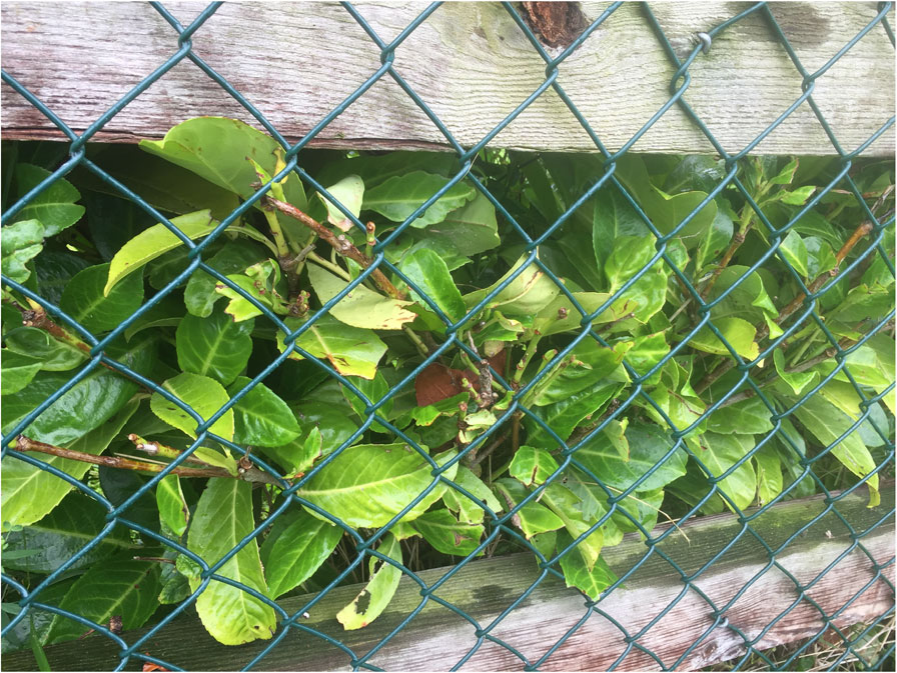
Laurel hedge bounding the field where the animals appeared to have chewed a number of the leaves

**Fig. 2:**
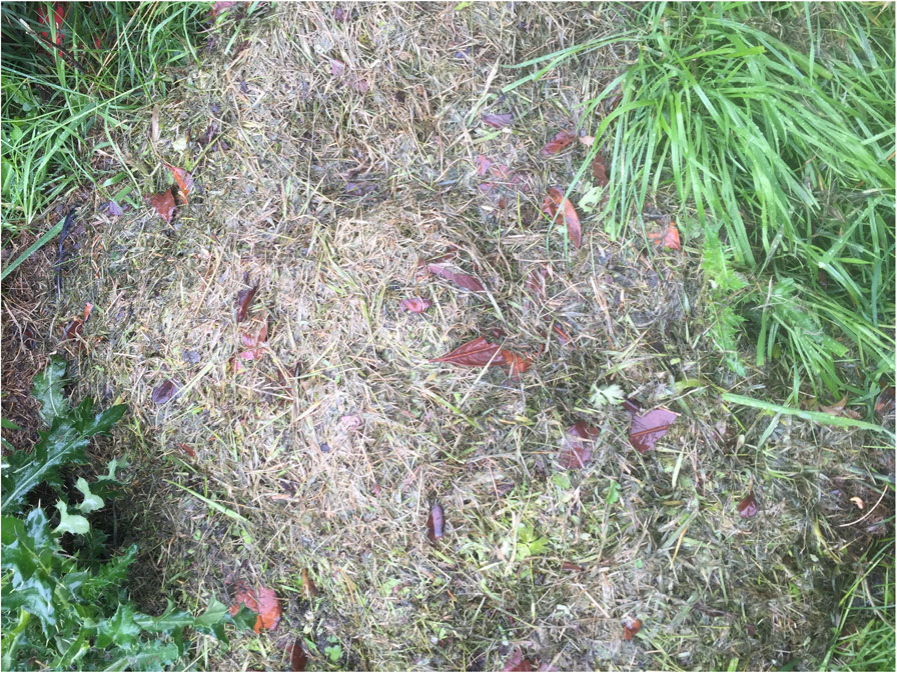
Lawn clippings with dead leaves were in several locations at the boundaries of the field

Re-examination of the field identified a laurel hedge approximately 60 m in length bounding a garden fence on one side of the field (Fig. 1). Several laurel leaves appeared to be chewed. In addition, there were multiple piles of lawn / garden clippings with rotting laurel leaves (Fig. 2), noted in the field.

During the farm visit the surviving 28 calves were examined; one was moribund, and two were recumbent and hyperaemic mucous membranes were noted in these three animals. Clotted, serum and EDTA blood samples were collected from these three animals. The samples tested negative for Malignant Catharral Fever; aetiological agent Ovine Herpesvirus-2 (OHV2), Bluetongue (*Orbivirus*) and Bovine Viral Diarrhoea (BVD - *Pestivirus*). Significant titres were recorded for *Salmonella* Dublin. There was no history of recent Salmonella vaccination.

Haematology parameters indicated a neutropaenia in two animals and a neutrophilia in the third.

Several calves had an increased respiratory rate. The body condition of the majority of the animals was poor, the farmer reporting that body condition had deteriorated dramatically since the onset of clinical signs. It was requested that additional animals were submitted for post mortem examination.

### Post mortem findings

#### Weanling 1 (submitted more than 24 h post mortem)

**Fig. 3:**
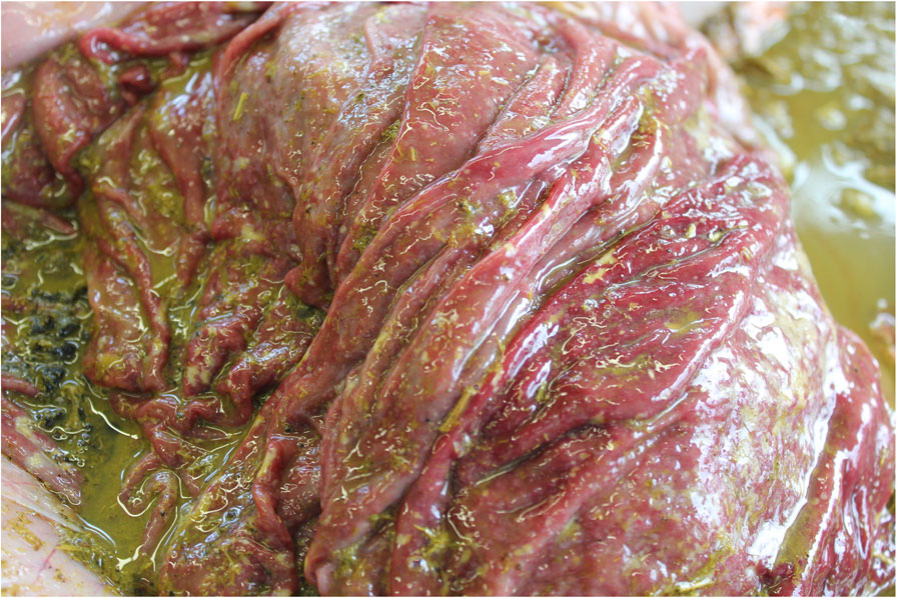
Gross abomasal pathology in weanling 1 showing a diffusely hyperaemic and oedematous abomasum, with multifocal to coalescing irregular pale white foci

Gross examination of weanling 1 showed the abomasum to be diffusely hyperaemic and oedematous, with multifocal to coalescing irregular pale white foci (Fig. 3). There were some petechial haemorrhages affecting the thymus and there was some fibrin in the pericardial sac. There were discrete localised areas of consolidation in the lungs. There were watery green contents throughout the small and large intestines. No abnormalities were detected in the rumen content.

Main laboratory results included culture of *Escherichia coli* from multiple organs (lung, intestine and abomasum). The lung also tested BRSV positive by real-time multiplex Taqman® RT-PCR. There was a strongyle egg count of 150 eggs per gram of faeces. The rumen fluid pH, liver copper and cobalt, renal cortex lead and selenium concentration and ocular fluid nitrate, calcium and magnesium concentrations were within normal limits. *Clostridium perfringens* DipFit™ rapid diagnostic tests on intestinal contents were negative.

The main histopathological findings were mild interstitial pneumonia, mild multifocal chronic interstitial nephritis, chronic active enteritis and non-specific reactive hepatitis. Autolysis impaired interpretation of some tissues and prohibited interpretation of others.

#### Weanling 2: (submitted approximately 24 h post mortem)

Main Gross Post Mortem Finding: There were several partially digested laurel leaves in the ruminal contents. There was a severe abomasitis with erosions/ulcers and severe diffuse thickening and hyperaemia of the abomasal mucosal surface. Multifocally the lungs were heavy and congested, and the heart contained bright red coloured clots.

Significant laboratory results on weanling 2 included an extremely high strongyle count (> 4000 eggs per gram). Respiratory pathogens detected on PCR included BRSV, *Histophilus somnus* and *Pasturella multocida*. *Escherichia coli* was cultured on the liver and intestine.

Rumen pH, liver copper and cobalt concentration, renal cortex lead and selenium concentration, and ocular nitrate, calcium and magnesium concentrations were within normal limits. *Clostridium perfringens* DipFit™ rapid diagnostic tests on intestinal content were negative.

Histopathological examination of the abomasum revealed a severe sub-acute multifocal abomasitis with ulceration, necrosis and vasculitis with thrombosis. Examination of the kidney revealed multifocal vasculitis with thrombosis and a chronic mild interstitial nephritis. In addition, examination of the small intestines revealed foci of necrosis with a neutrophilic infiltrate, and moderate numbers of lymphocytes and macrophages were seen in the submucosa; multifocal necrotising enteritis.

### Cyanide testing

Given the evidence of access to the laurel hedges, the plausible timeline and the evidence of plant material in one rumen content, cyanide was considered as a possible contributory factor in the farm fatalities. Arrangements were therefore made to test samples for cyanide concentration. Collection of liver and muscle samples for cyanide testing post mortem is recommended within four and twenty hours after death respectively [10]. As both weanlings had been dead greater than 24 h prior to their submission for post mortem it was decided to collect fresh *ante mortem* whole blood samples from a random selection of cohort animals. Blood samples were collected from seven animals directly into heparinised and K2EDTA tubes. The tubes were filled to the top to minimise their air content and refrigerated as soon as possible. On another unconnected holding two bovine blood samples were collected directly into heparinised tubes to serve as negative controls. The samples were shipped immediately to Toxlab, Paris, France for analysis by LC-Fluorimetry.

### Cyanide test results

Cyanide levels greater than 1 mg/litre are typically consider toxic in mammals and birds [7]. Of the seven samples one animal recorded a reading of 3.4 mg/litre. This animal subsequently died. Cyanide was not elevated above the normal limits in the remaining six samples. Five of these animals remain in the herd.

## Discussion and conclusion

Internationally, group outbreaks of cyanide toxicity have previously been reported following ingestion of amongst others reed sweet grass, vetch hay, apricot kernels and red crumbweed [15–18]. This group outbreak of suspect cyanide poisoning, due to consumption of laurel, is, to the author’s knowledge, the first such reported case report in cattle in Ireland. Although the gross, histopathological and laboratory findings suggest a possible role for concurrent disease in the fatalities recorded in this case the identification of laurel leaves in rumen content, the high cyanide level in blood from a cohort (taken a number of days after the initial exposure) and the reports of mucous membrane cyanosis by the attending veterinarian and the rapid onset and intense course of the clinical signs is compelling evidence of a role for cyanide toxicity in the deaths of these animals.

The diarrhoea reported in this case was also described in goats which became comatose in a case of cyanide toxicity associated with the ingestion of crab apples [19]. The increased volume of pericardial fluid and haemorrhages noted in this case were also reported by McKenzie et al. (2007), and reddening of the abomasum was described by Sharmann et al.. (1976). Indeed, a report by Nicholson [20], indicated that laurel contains essential oils similar to turpentine that can cause irritability of the mucosa which may partly explain gross findings on the abomasum. However, additional PM findings and laboratory results in this case, suggest there could have been multiple factors involved in this large herd mortality event. These may have complicated/exacerbated the affects of laurel ingestion. It may be reasonable to suggest that *Salmonella* could have played a contributory role in development of the abomasitis, vasculitis and in the non-specific histopathological findings in the intestines in both weanlings. Significant *Salmonella* titres were detected in blood from cohort animals on the farm, however *Salmonella* was not detected on selective culture media, although it should be noted that antibiotic therapy had been administered. Equally the high strongyle count in weanling 2 would have compromised the mucosal lining of the abomasum and may have allowed the development of a bacteraemia/septicaemia, which may be responsible for observed vasculitis seen in the abomasum and kidneys. Viral causes of vasculitis (MCF, BVD, BT) were investigated and not detected, and fungi were not observed histopathologically. A hypersensitivity reaction was also considered as a possible aetiology of the vasculitis and, although it cannot be discounted, the high strongyle egg count or a potential salmonellosis seems most plausible. While 22 animals died within two days of exposure to the laurel hedge, a further 14 deaths were recorded over a 12-day period. These concurrent diseases may account for the extended period over which animals continued to die. Although not reported in the literature, the possible stress associated with sub-lethal consumption of laurel may have exaggerated the pathology of pre-existing sub-clinical diseases present in this herd, eventually leading to death. Unfortunately, although additional carcasses were requested for post mortem examination, only two carcases were submitted. The outbreak occurred during a period of COVID-19 restrictions in Ireland which limited sample submission, so it is not known if other animals affected had similar pathology.

Unusually, lethal cyanide levels were detected in an animal that survived a week after suspected laurel ingestion. This prolonged period of survival is not typical of cyanide toxicity and may indicate that under certain circumstances the course of the disease may be prolonged. Robb et al. [21] and Wilson et al. [22] previously reported a delay in the onset of clinical signs even in the absence of continued plant consumption. It has been suggested that the digestibility levels of the cyanide containing material may delay the onset of the disease [10]. Laurel leaves are generally considered tough and robust. Potentially this may have prolonged the time to digestion and release of HCN. Nicholson [9] suggested that dry rumen contents can delay the release of HCN by rumen flora until the animals drink water, but this is unlikely to be relevant in this situation one week later. The amount of plant consumed may also have impacted the speed of development of clinical signs. The minimum lethal dose of cyanide is variable depending on the rate of intake [23, 24]. Ruminants are typically more susceptible to cyanide toxicity due to higher ruminal pH compared to monogastric species [10]. However, if the ingestion of lawn clippings had resulted in acidosis, as initially suspected, it may have altered the ruminal pH and the speed of HCN conversion. Additionally, low levels of cyanide can be detoxified in the liver. An important but minor pathway of detoxification includes the combination of cyanide with hydroxy-cobalamin (vitamin B12) to yield cyanocobalamin (vitamin B12) which is then excreted in the urine [8]. In advance of identifying cyanide as the potential causative agent, vitamin B complex injections were administered by the attending veterinarian, and hydroxy-cobalamin has been used as a cyanide antidote [7, 8]. This potentially slowed progression of clinical signs, or indeed possibly aided recovery in the surviving animals that may have ingested sub-lethal doses.

Failure of animals to recognise and avoid toxic plants [17], and unknowingly offering a toxic feed as a primary part of the diet [15, 16], are previously reported factors in group cyanide toxicity cases. In contrast, in the current situation the affected animals were both familiar with the area and had access to a non-cyanogenic feed source (grass). The field had been grazed by this cohort and other groups in previous years without the ingestion of the laurel hedge. The reason why the animals ingested laurel in this situation is not known. Payne et al.. (2014) indicated contributory factors in plant poisonings including the relative non-selective grazing behaviours of cattle and the inquisitive nature of younger animals in particular. Regardless of the underlying cause, the farmer was advised to prevent future access to the laurel hedges.

While there were potentially multiple agents at play, the high fatality rate serves as a timely reminder to include plant poisoning as a differential diagnosis when dealing with large numbers of rapid fatalities. Examination of fields for potentially toxic plant material is recommended especially following hedge-cutting and after storms that could potentially lead to fallen leaves/ trees, allowing easy access for ruminants to consume. Failure to thoroughly examine rumen contents and collect a detailed history in this case, could easily have allowed the involvement of cyanide toxicity to be missed, and death attributed to other causes. In cases of individual or group fatalities history-taking is important and recent entry to new grazing areas or any potential dietary change should be thoroughly investigated. Education of well-meaning neighbours on the potential risk of offering lawn clippings, or other garden waste to farm animals is also advisable.

## Data Availability

not applicable.

## Abbreviations

DAFM: Department of Agriculture, Food and the Marine
RVL: Regional Veterinary Laboratory
